# The anticancer mechanism investigation of Tanshinone II_A_ by pharmacological clustering in protein network

**DOI:** 10.1186/s12918-018-0606-6

**Published:** 2018-10-29

**Authors:** Yan-feng Cao, Shi-feng Wang, Xi Li, Yan-ling Zhang, Yan-jiang Qiao

**Affiliations:** 1 School of Chinese Materia Medica, Beijing University of Chinese Medicine, Beijing, 100102 China; 2 Beijing University of Chinese Medicine, No. 11, Bei San Huan Dong Lu, Chaoyang District, Beijing, 100029 China

**Keywords:** Tanshinone II_A_, Antineoplastic agents, Network pharmacology, Pharmacological clustering

## Abstract

**Background:**

Cancer is the second most common cause of death globally. The anticancer effects of Tanshinone II_A_ (Tan II_A_) has been confirmed by numerous researches. However, the underlying mechanism remained to be integrated in systematic format. Systems biology embraced the complexity of cancer; therefore, a system study approach was proposed in the present study to explore the anticancer mechanism of Tan II_A_ based on network pharmacology.

**Method:**

Agilent Literature Search (ALS), a text-mining tool, was used to pull protein targets of Tan II_A_. Then, pharmacological clustering was applied to classify obtained hits, the anticancer module was analysed further. The top ten essential nodes in the anticancer module were obtained by ClusterONE. Functional units in the anticancer module were catalogued and validated by Gene Ontology (GO) analysis. Meanwhile, KEGG and Cell Signalling Technology Pathway were employed to provide pathway data for potential anticancer pathways construction. Finally, the pathways were plotted using Cytoscape 3.5.1. Furthermore, in vitro experiments with five carcinoma cell lines were conducted.

**Results:**

A total of 258 proteins regulated by Tan II_A_ were identified through ALS and were visualized by protein network. Pharmacological clustering further sorted 68 proteins that intimately involved in cancer pathogenesis based on Gene Ontology. Subsequently, pathways on anticancer effect of Tan II_A_ were delineated. Five functional units were clarified according to literature: including regulation on apoptosis, proliferation, sustained angiogenesis, autophagic cell death, and cell cycle. The GO analysis confirmed the classification was statistically significant. The inhibiting influence of Tan II_A_ on p70 S6K/mTOR pathway was revealed for the first time. The in vitro experiments displayed the selectivity of Tan II_A_ on HeLa, MDA-MB-231, HepG2, A549, and ACHN cell lines, the IC_50_ values were 0.54 μM, 4.63 μM, 1.42 μM, 17.30 μM and 204.00 μM, respectively. This result further reinforced the anticancer effect of Tan II_A_ treatment.

**Conclusions:**

The current study provides a systematic methodology for discovering the coordination of the anticancer pathways regulated by Tan II_A_ via protein network. And it also offers a valuable guidance for systematic study on the therapeutic values of other herbs and their active compounds.

**Electronic supplementary material:**

The online version of this article (10.1186/s12918-018-0606-6) contains supplementary material, which is available to authorized users.

## Background

Tanshinone II_A_ (Tan II_A_) is a phenanthrene-quinone extracted from a medicinal herb Danshen, *Salvia miltiorrhiza* Bunge. The herb has been used in Traditional Chinese medicine (TCM) practice for over a millennium. It possesses efficacy of promoting blood circulation and preventing blood stagnation based on years of clinical observation. Chinese medicine practitioners have applied herbs with such function to treat cancerous diseases based on detectable clinical assessments [1–3] even before the modern concept of cancer was defined.

Nowadays, cancer is the second most common cause of death in developed and several developing countries. The conventional therapies, radiotherapy and chemotherapy, have showed their limitations and drawbacks, for instance, severe side-effects, intolerance and increasing resistance. Hence the need for developing a series of new anticancer drugs is growing. Phytochemical compounds are a promising source, as groups of plant derivatives, such as paclitaxel, trabectedin, and vincristine, have already been developed into therapeutic drugs. The urgent need for novel anti-tumour agents hastens the researches on anticancer activity of Danshen and its major active component. Tan II_A_ has been considered to possess similar function as its parent herb. In modern medicinal studies, it has been reported to exhibit a variety of pharmacological activities, including but not limited to anti-inflammatory, anti-oxidative, anti-atherosclerosis, and anticancer [4, 5]. The anticancer activity of Tan II_A_ has attracted numerous interests in the past decade. The anticancer effects and potential mechanisms of Tan II_A_ have been studied extensively in various cancer cell lines and showed promising activity via in vivo experiments [6, 7] and clinical studies [8].

The large number of researches on anticancer effect of Tan II_A_ provided a great opportunity for effective data mining and network pharmacological study on this subject. Network pharmacology, based on network biology and systems biology, has been favoured by TCM researchers. The network pharmacology shares same guiding theory with TCM practice which is ‘parts can be understood only in its relation to the whole [9]’. In the present case, the regulatory effects of Tan II_A_ on its distinctive targets can provide detectable therapeutic outcomes only when they are added-up. TCM network pharmacology integrates TCM theory with molecular networks that focuses on the systematic effects of drug targets on the biological network [10]. It offers a novel researching approach for mapping the anticancer mechanisms of Tan II_A_ and identifying potential protein targets that coordinated to produce synergetic effect.

With the exponential expansion of publications, literature mining became a critical tool for a labour-saving secondary study based on the primary experiment data. Text mining by a Cytoscape plug-in, Agilent Literature Search (ALS), retrieved results from various reliable data resources such as PubMed and provided evidence-based outcomes for further manual inspection. This approach was similar to the ‘lightweight’ approach to text mining which proved robust and sustainable [11]. KEGG has been characterized as a third-generation approach [12] in pathway analysis. It offers valuable information on the interaction of proteins for the present system study. Pharmacological clustering, on top of topological analysis has been applied in other studies, was utilized in the present network study. This clustering method was developed based on National Center for Biotechnology Information (NCBI) oncology database, it generated modules based on the function of individual protein instead of topological features of the network. The modules derived from pharmacological clustering were intimately connected to the function, the proteins in the same module were more likely to produce similar pharmacological effects in response to a given chemical entity.

In addition, to evaluate the potential of regulatory effects of Tan II_A_ on various cancer types, the inhibitory effects were tested by in vitro cell proliferation experiments with several cell lines. Breast cancer, lung cancer, kidney cancer and cervical cancer were among the most common cancers [13, 14], hence the sensitivity and specificity of these cancers to Tan II_A_ treatment was assessed on the representative cell lines of these cancers.

The present work aimed to understand the antineoplastic action of Tan II_A_ in a systematic and comprehensive angle. Therefore, network pharmacological approaches combing in vitro experiments were applied to construct a reliable systematic network to illustrate the mechanism and would also valuable for further research on the therapeutic values of Chinese herbal products.

## Results

### A network of all proteins regulated by Tan II_A_

**Fig. 1:**
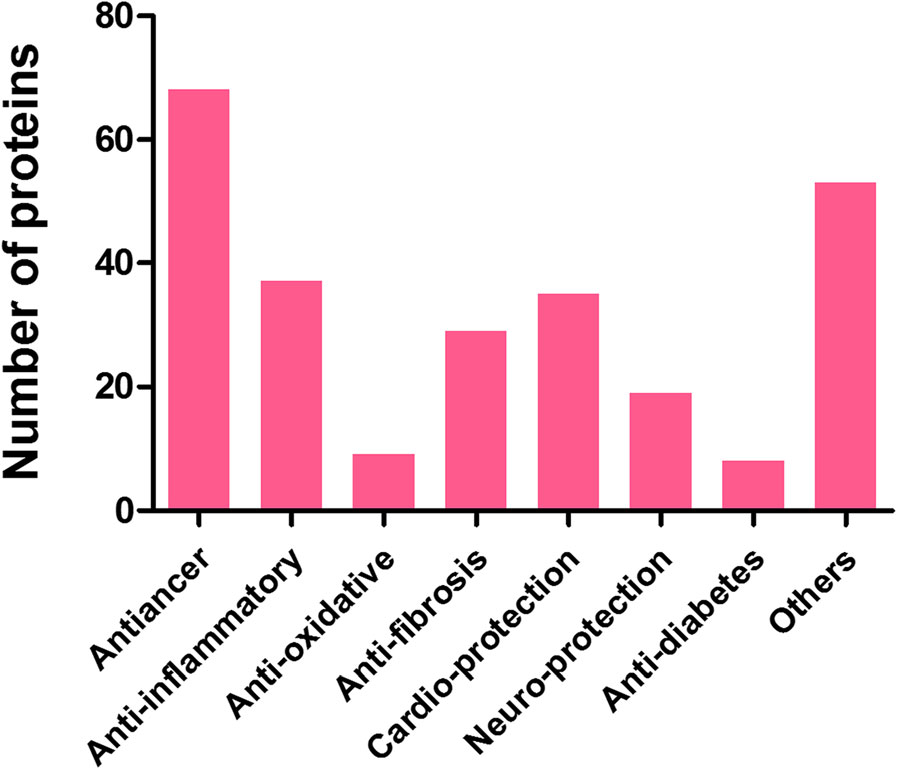
Pharmacological clusters and number of proteins regulated by Tan IIA. Sorted by pharmacological clustering method based on function of proteins

The network constructed by ALS data mining contained all proteins under the influence of Tan II_A_ treatment. Target proteins were identified by official symbol and other aliases, the abbreviations used in research papers could accidentally coincide with them. Therefore, checks against supporting evidences for each hit have been done to ensure correct protein identification, false positives were processed. For example, 1. aliases: search hit “pkb” and “akt1” both stand for “AKT serine/threonine kinase 1”, “jun” and “mapk8” for “c-Jun N-terminal kinases” they were merged as node “Akt” and “JNK” respectively; 2. false identification, “FBS” (Fetal bovine serum) in original research paper [15] was falsely identified as “F-box protein 8”, the node was subsequently deleted. In total, 396 nodes were retrieved, among which 258 proteins were regulated by Tan II_A_. The functions of proteins were obtained from NCBI databases and other recent researches. The pharmacological activities of Tan II_A_ could be classified into seven major aspects, namely anticancer, anti-inflammatory, anti-oxidative, anti-fibrosis etc. (Fig. 1).

### The identification of anticancer network of Tan II_A_ through pharmacological cluster

**Fig. 2:**
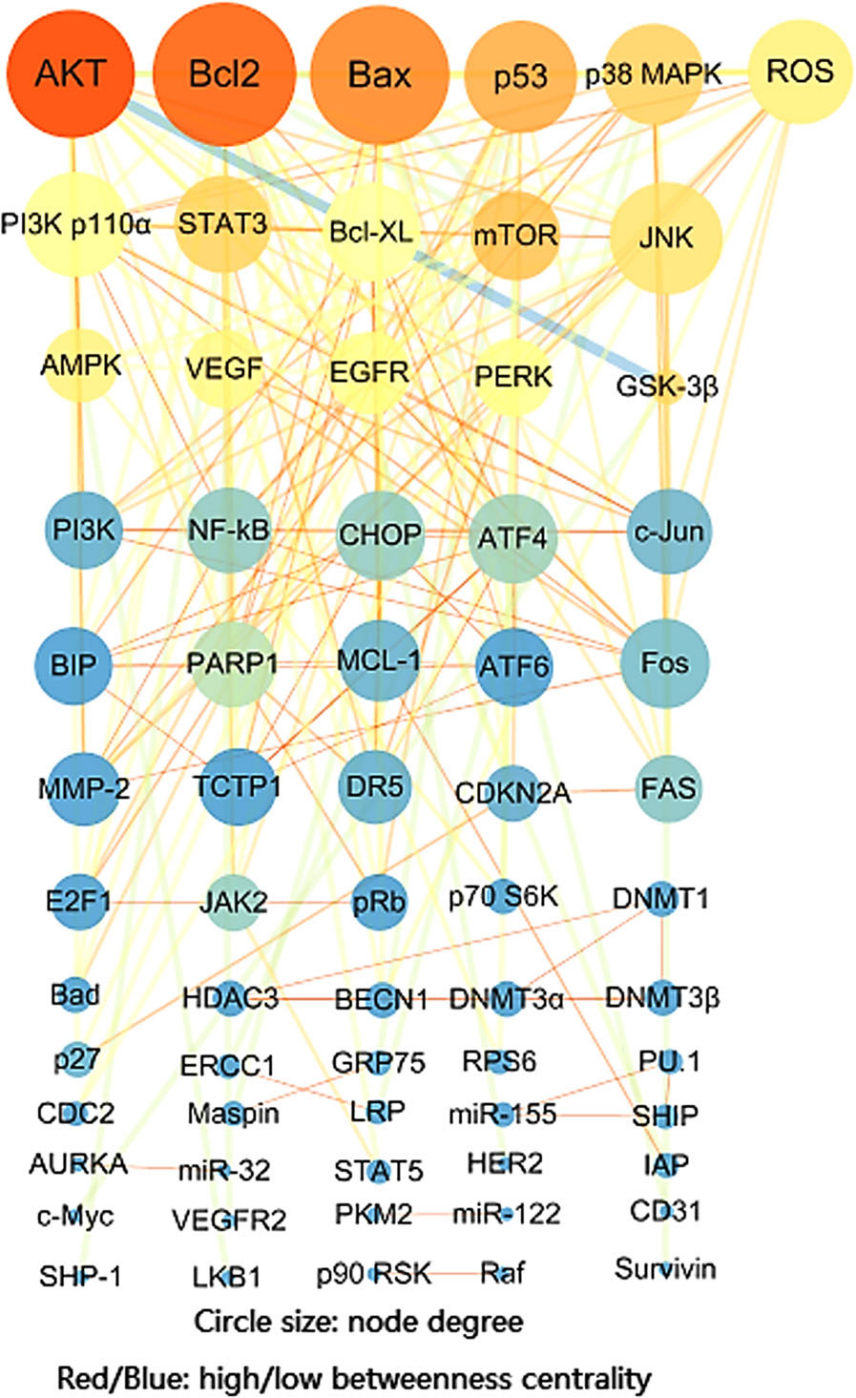
Tan II_A_ targets network on cancer created by pharmacological clustering. Analyzed by NetworkAnalyzer. The colour of the node change from blue to red with increased betweenness centrality. The circle size was proportional to the degree of the node. The edge thickness showed degree of connection between two connecting nodes

**Fig. 3:**
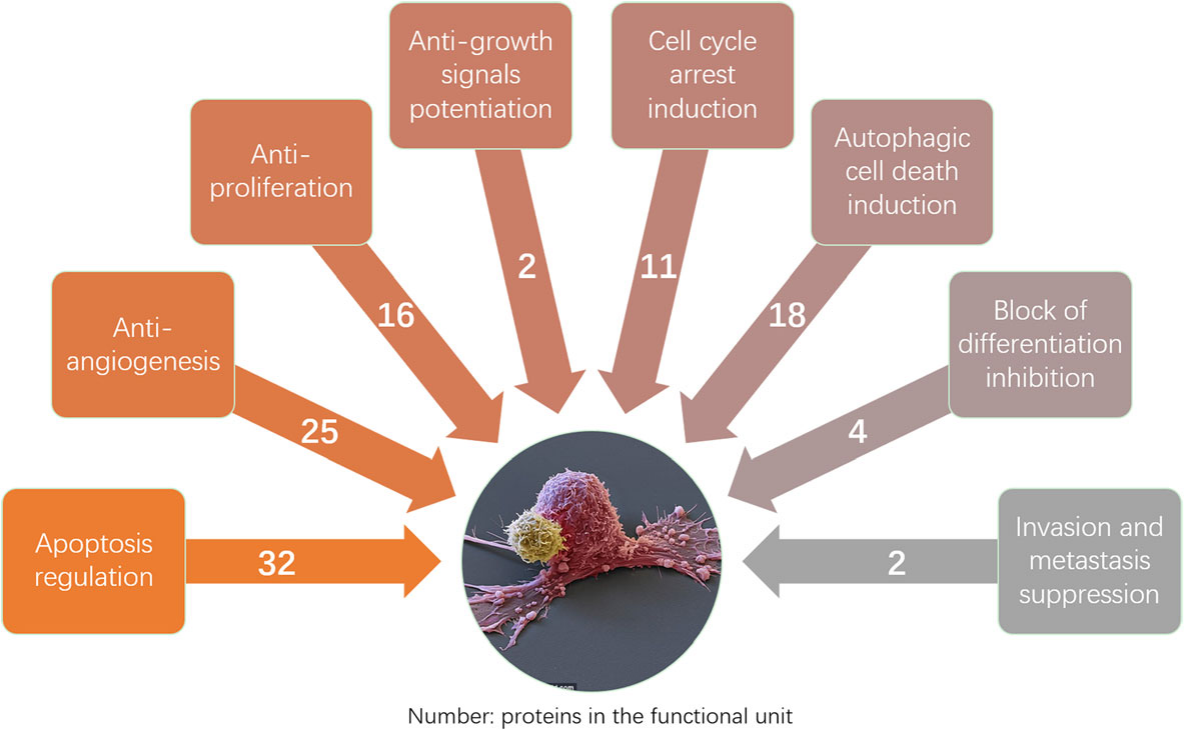
The functional units of Tan II_A_ and number of proteins involved in the regulation of cancer phenotypes. Sorted according to current universally recognized major characteristics of cancer [18]

The pharmacological cluster on cancers was illustrated (Fig. 2). The node degree distribution followed power law P(k) = 16.574×^-0.848 (correlation:0.914, R-square:0.800), which indicated that the anticancer network was a scale-free network. A total number of 8 Tan II_A_ anticancer functional units classified upon the characteristics of cancer they targeted were illustrated [20] (Fig. 3). The present study focused on clusters with a size above 10. The GENEONTOLOGY (GO) enrichment analysis confirmed the clustering results, the biological processes discovered by GO analysis were in consistent with the units (Table 1). All results had *P* value and FDR (False Discovery Rate) lower than 0.05, which indicated they were statistically significant. In topological analysis, the degree of a node was the number of connections it has to other nodes, a node with higher betweenness centrality would have more control over the network [16, 17]. In the present pharmacological cluster, the top 10 nodes identified by CytoHubba were Bcl-2, BCL2 associated X (Bax), B-cell lymphoma-extra large (Bcl-XL), DNA damage inducible transcript 3 (CHOP), induced myeloid leukemia cell differentiation protein (MCL1), translationally controlled tumour protein (TCTP), heat shock protein family A member 5 (BIP), protein kinase B (PKB) also known as Akt, phosphoinositide 3-kinase (PI3K) and Fos proto-oncogene, AP-1 transcription factor subunit (Fos).

**Table 1:** Validation of networks

NETWORK	FIGURE	GO BIOLOGICAL PROCESS	P VALUE	FDR (FALSE DISCOVERY RATE)
APOPTOSIS REGULATION	4	apoptotic process	2.02E-21	2.43E-18
ANTI-ANGIOGENESIS	5	angiogenesis	1.23E-05	2.74E-02
ANTI-PROLIFERATION	6	cell proliferation	4.56E-04	1.63E-02
CELL CYCLE ARREST	7	regulation of cell cycle	9.87E-06	2.90E-03
AUTOPHAGIC CELL DEATH	8	regulation of autophagy	4.44E-08	6.93E-04

### Apoptosis regulatorypathway analysis of Tan II_A_

**Fig. 4:**
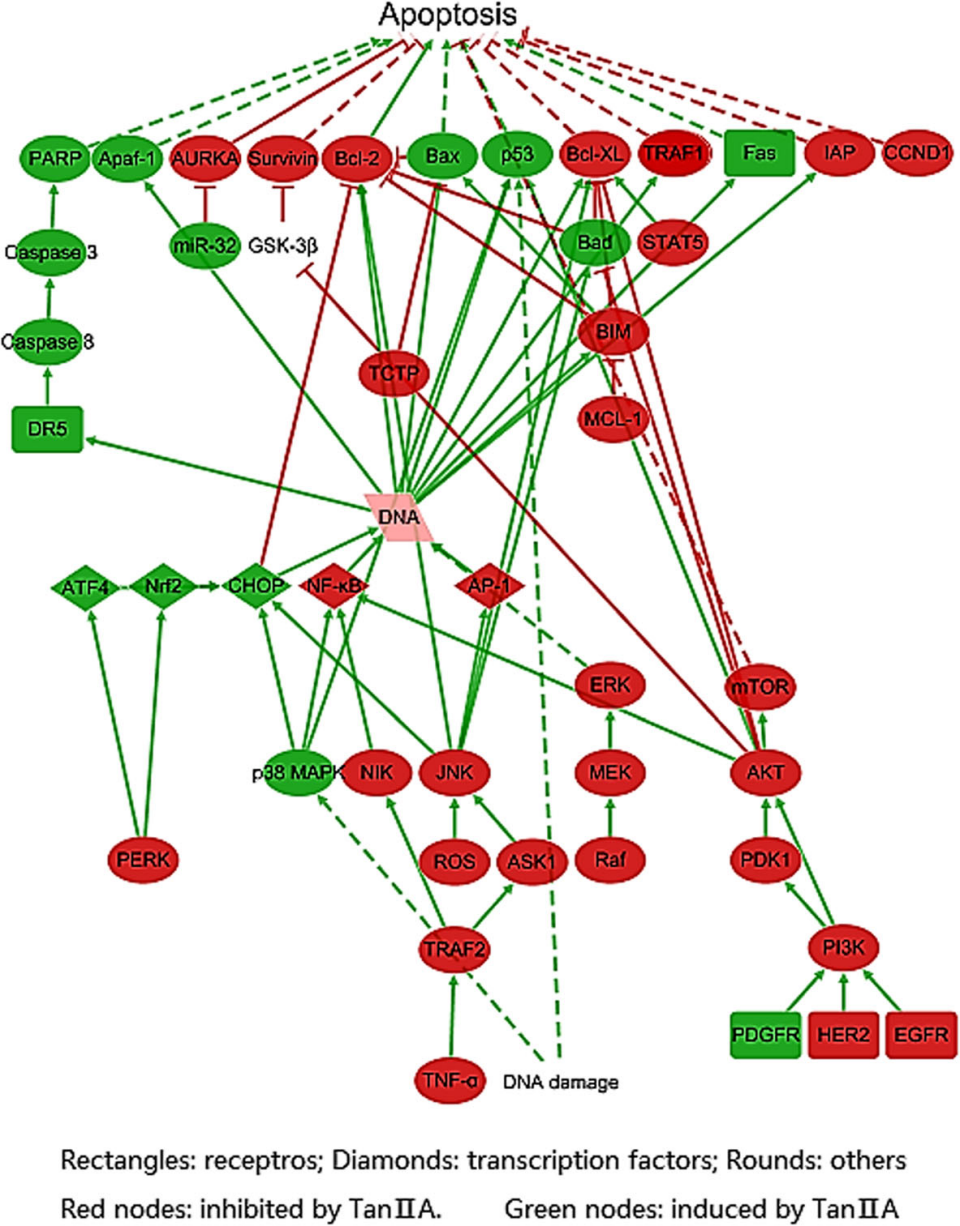
The regulation of Tan II_A_ on pathways in apoptosis process of cancer development. Constructed based on information from KEGG “Pathways in Cancers (has 05200)” and “Apoptosis (has 04210)”, and CST pathways “Regulation of Apoptosis Overview”and “PI3 Kinase/Akt Signalling”, visualized by Cytoscape 3.5.1

Resistance to apoptosis, an aspect of uncontrolled proliferation which is a distinguishing characteristic of cancer. A functional unit on pathways which regulate apoptosis process was constructed based on literatures referred by ALS (Fig. 4, Additional file 1). The network demonstrated that several signalling pathways were inhibited by Tan II_A_ simultaneously. On the contrary, Tan II_A_ up-regulated p38 mitogen-activated protein kinase (MAPK) signalling. Among these proteins, AKT protein in the PI3K/AKT pathway and c-jun N-terminal kinase (JNK) had the highest degree of 7. Moving along the pathway network, Tan II_A_ elevated the protein expression of transcription factors: activating transcription factor 4 (ATF4), nuclear factor (erythroid-derived 2)-like 2 (Nrf2) and CHOP; whereas, it decreased the expression of nuclear factor kappa B (NF-κB) and AP-1. CHOP and NF-κB had the highest degree of 4. The results also showed, in short, that Tan II_A_ promoted apoptosis by inhibiting the expression of anti-apoptotic regulators and inducing pro-apoptotic regulators. The degrees (7) of anti-apoptotic regulators, Bcl-2 and Bcl-xL, were the highest. The network also revealed that Tan II_A_ up-regulated proteins which induce apoptosis such as tumour suppressor p53 [19], Bax [20–23] and poly(ADP-ribose) polymerase 1 (PARP1) [22] via both caspase-dependent pathway and caspase-independent pathway. Meanwhile, Tan II_A_ downregulated inhibitor-of-apoptosis protein (IAP) [24], mediator of the anti-apoptotic signals TNF receptor associated factor (TRAF), cyclin D1 (CCND1) [25] and survivin [26] expression by suppressing PI3K/Akt [27, 28], mitogen-activated protein kinase kinase (MEK)/ extracellular signal–regulated kinase (ERK) [29–31], Janus Kinase (JAK) / Signal Transducer and Activator of Transcription (STAT) [25, 32] and mTOR signalling pathways. Several membrane proteins erb-b2 receptor tyrosine kinase 2 (ERBB2) also known as HER2 [33], epidermal growth factor receptor (EGFR) [28], platelet derived growth factor receptor (PDGFR), TNF receptor superfamily member 10b (DR5) [30] and Fas cell surface death receptor (Fas) [34] were also regulated by Tan II_A_. Moreover, Tan II_A_ increased production of reactive oxygen species which increases oxygen stress in cell and lead to apoptotic cell death.

### Anti-angiogenesis pathway analysis of tan II_A_

**Fig. 5:**
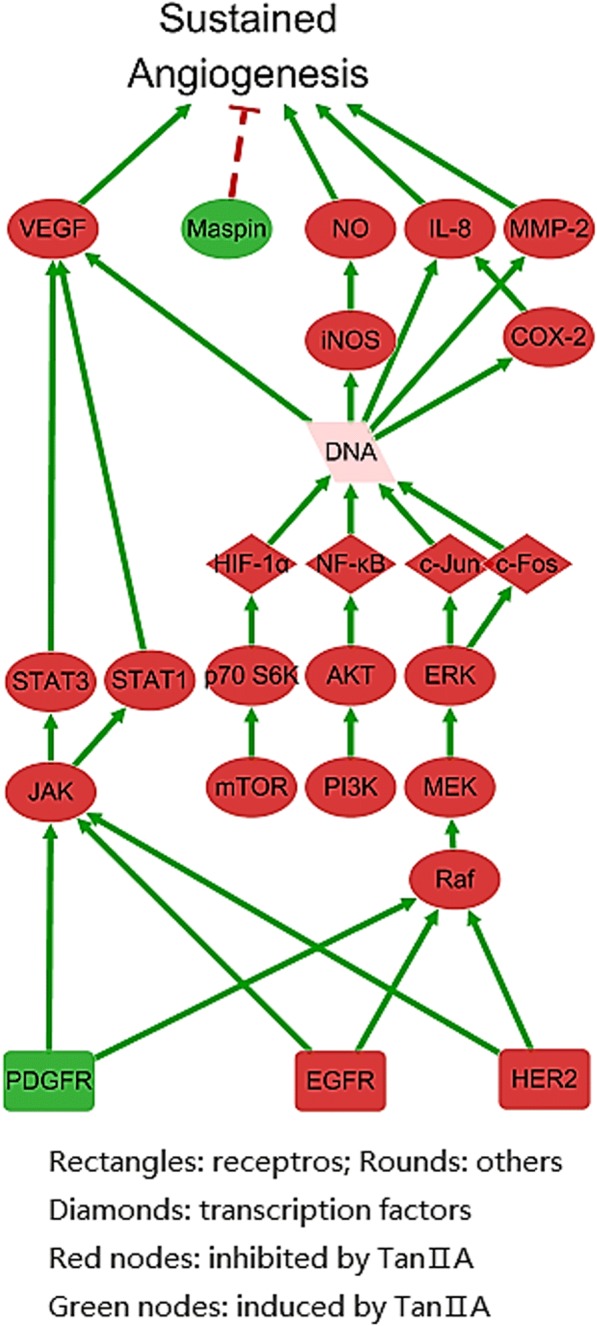
The regulation of Tan II_A_ on pathways in angiogenesis process of cancer development. Constructed based on information from KEGG “Pathways in Cancers (has 05200)” and CST pathways “Angiogenesis” and “mTOR Signalling” visualized by Cytoscape 3.5.1

Sustained angiogenesis is another pathological factor that contributes to uncontrolled proliferation of cancer cells. The pathway network illustrated a part of the systematic regulatory effect of Tan II_A_ on cancer (Fig. 5). It showed that Tan II_A_ produced coordinated regulation on sets of functionally related proteins and resulted in an overall anti-angiogenesis effect. Tan II_A_ inhibited four signalling pathways: PI3K/AKT, JAK/STAT, mTOR and MEK/ERK which in turn down-regulated the expression of three transcription factors: AP-1, HIF-1αand NF-κB. As a result, vascular mediator nitric oxide (NO), matrix metalloproteinase 2 (MMP2), vascular endothelial growth factor (VEGF), cyclooxygenase – 2 and pro-inflammatory cytokine interleukin-8 (IL-8) were inhibited. Among which, VEGF had the highest degree of 6. Meanwhile, mammary serine protease inhibitor (maspin) was up-regulated.

### Pathway analysis of anti-proliferation effect on Tan II_A_

**Fig. 6:**
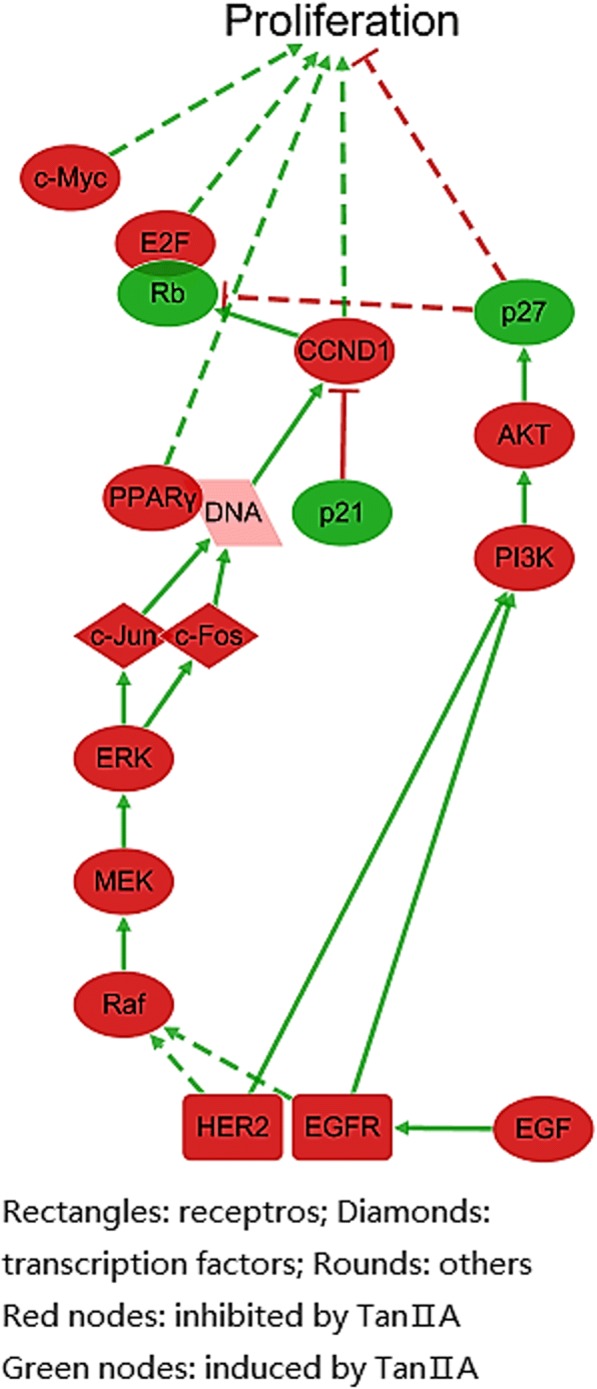
The regulation of Tan II_A_ on pathways in cancer cell proliferation. Constructed based on information from KEGG“Pathways in Cancers (has 05200)” and CST pathways “PI3 Kinase/Akt Signalling” and “MAPK/ERK in Growth and Differentiation”, visualized by Cytoscape 3.5.1

As mentioned above, uncontrolled proliferation is a major characteristic of cancer cell, the inhibitory activity of Tan II_A_ on cancer cell proliferation was important to its overall anticancer activity. The pathways of the anti-proliferation mechanisms of Tan II_A_ was built on evidences provided by ALS (Additional file 1) and information from KEGG and CST pathways (Fig. 6). The diagram showed Tan II_A_ inhibited two receptors: epithelial growth factor receptor (EGFR) and receptor tyrosine-protein kinase erbB-2 (HER2), it supressed two signalling pathways and down-regulated the expression of proteins that potentiate proliferation such as CCND1, MYC proto-oncogene (c-Myc) [35]. Tan II_A_ induced interferon alpha-inducible protein 27 (p27) which negatively regulates proliferation.

### Pathway analysis of carcinoma cell cycle arrest effect of Tan II_A_

**Fig. 7:**
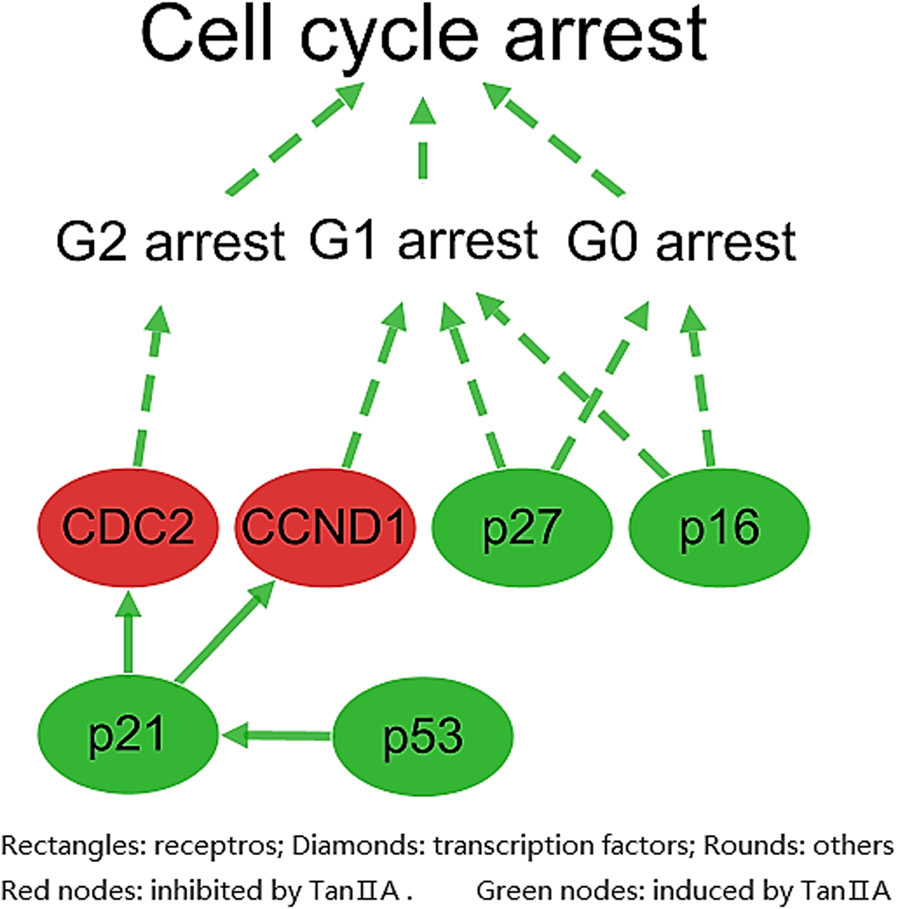
The regulatory effects of Tan II_A_ on cancer cell cycle arrest. Constructed based on information from KEGG“Pathways in Cancers (has 05200)” and CST pathways “Cell Cycle Control: G1/S Checkpoint”, visualized by Cytoscape 3.5.1

Other than uncontrolled proliferation, another significant features of cancer is dysregulation of cell cycle [36]. As one mechanism evolved in response to DNA damage is halting cell-cycle progression, any alteration would increase the risk of cancer developing [37]. The pathway network (Fig. 7) was shaped by supporting evidences (Additional file 1) submitted by ALS and information from KEGG and CST pathways. The pathway network revealed the overall inducible effects of Tan II_A_ on cell cycle arrest. Tan II_A_ activated phosphorylation of p27; it induced the expression of cyclin-dependent kinase inhibitor 2A (p16) and p53, tumour suppressors, and the expression of cyclin-dependent kinase inhibitor 1 (p21). On the other hand, Tan II_A_ inhibited the expression of CCND1 and the phosphorylation of cyclin dependent kinase 1 (CDC2).

### Pathway analysis of autophagic cell death induced by Tan II_A_

**Fig. 8:**
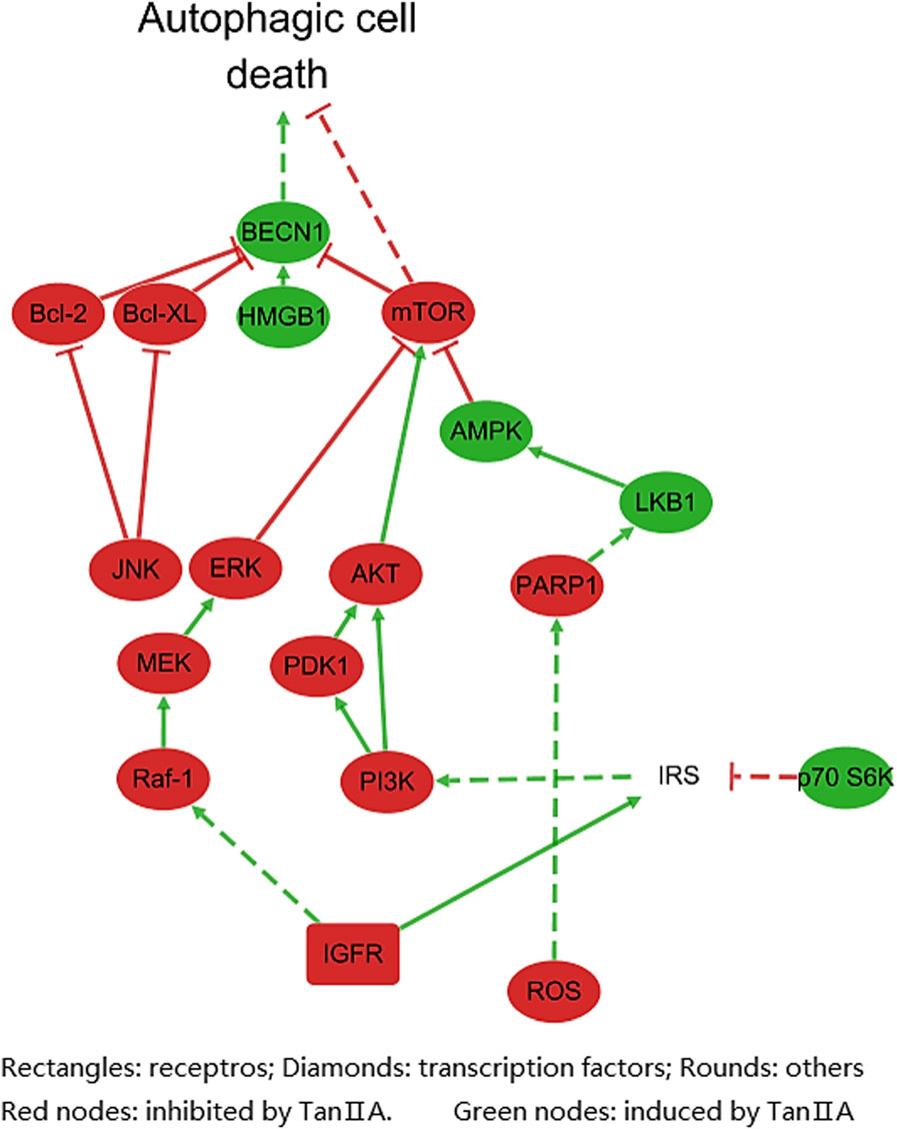
The regulatory effects of Tan II_A_ on autophagic cell death of cancer cells. Constructed based on information from KEGG“Pathways in Cancers (has 05200)” and“Autophagy (hsa04140)”, and CST pathways “Autophagy Signalling” and “mTOR Signalling”, visualized by Cytoscape 3.5.1

In addition to above anticancer mechanisms, induction of autophagic cell death by Tan II_A_ also contributed to its anticancer activity. Autophagic cell death is a type of regulated, programmed cell death which is essential to ensure normal cell function. Evidences suggested autophagic cell death mediate cancer elimination and that decreased autophagic activity is related to tumorigenesis [38]. Research data on autophagic cell death was referred in ALS supporting evidences, which offered profile for the pathway (Fig. 8). Tan II_A_ antagonized insulin-like growth factor 1 receptor (IGFR), and decreased production of ROS. Tan II_A_ induced p70 S6K which indirectly inhibited PI3K/AKT pathway. Meanwhile it suppressed PARP1 and MEK/ERK signalling pathways simutanousely. The regulation on these three pathways resulted in the inhibition of mTOR pathway. Thus induced autophagic cell death, as elevated expression of beclin-1 (BECN1) was observed. Moreover, inhibition of JNK pathway and Bcl-2 and Bcl-xL also contributed to the increase of BECN1.

### Inhibitory effects of Tan II_A_ on various carcinoma cell lines

**Fig. 9:**
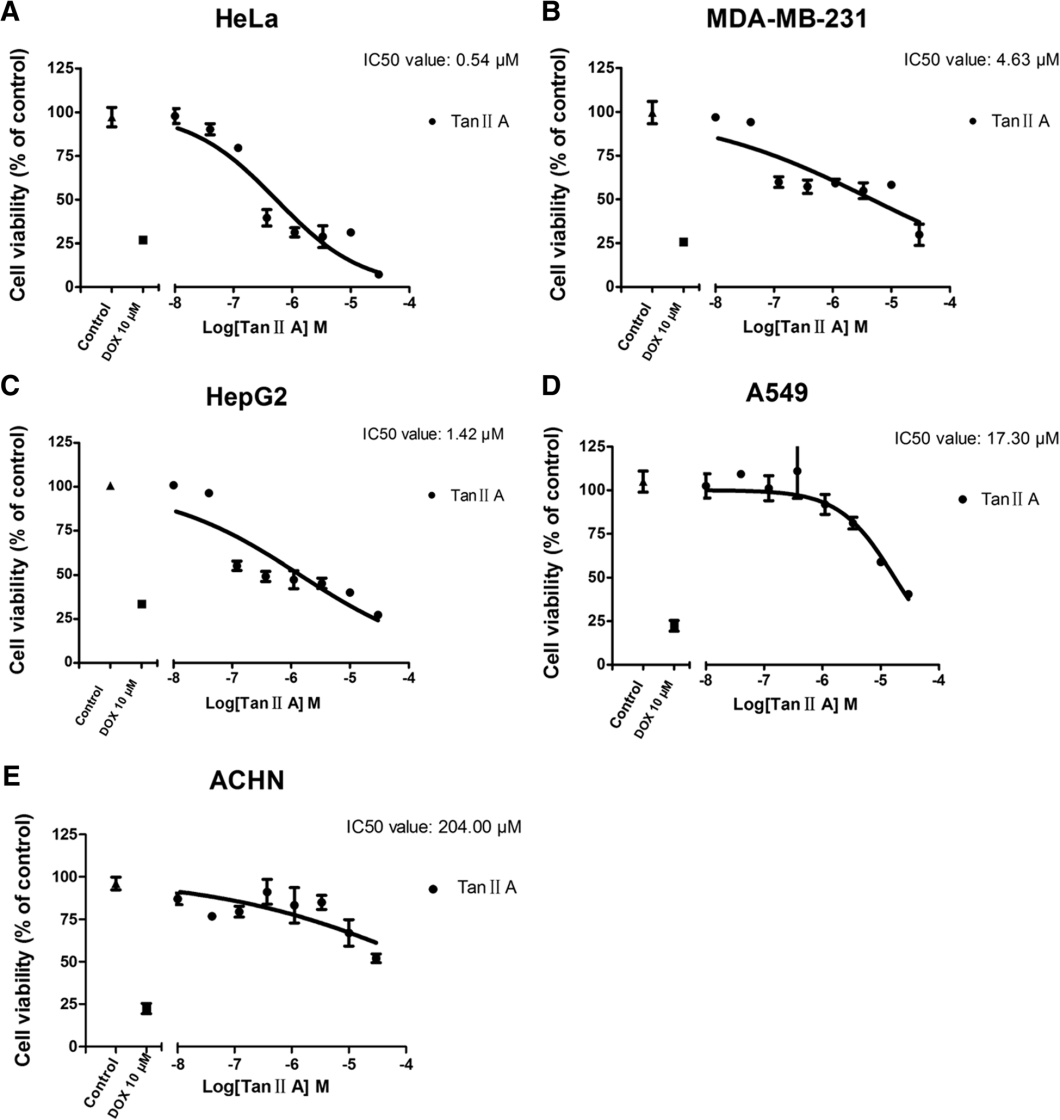
The inhibitory effects of Tan IIA on proliferation of various cell lines. (**a**-**e**) Dose response curve of Tan II_A_ treated Hela, MD-MB-231, HepG2, A549, ACHN cell lines. The cells were contineously incubated with test compound for 48 h. DMSO treated sample was determined as negative control. Doxorubicin (DOX, 10 μM) sample was defined as positive control. Data represented mean ± SEM, *n* = 4

To test the sensitivity and specificity of Tan II_A_ on various carcinoma cell lines, further assay on cell proliferation was conducted. Tan II_A_ exhibited dose-dependent inhibitory effects on HeLa, MDA-MB-231, HepG2, A549, and ACHN cells lines. The IC_50_ values were 0.54 μM, 4.63 μM, 1.42 μM, 17.30 μM, and 204.00 μM respectively (Fig. 9a-e). These figures demonstrated that Tan II_A_ exerted strong selectivity on HeLa cells, moderate effects on MDA-MB-231, HepG2, and A549 cells. While it merely exhibited mild influence on ACHN cells.

## Discussion

Cancer is one of the leading causes of morbidity and mortality worldwide and was responsible for around 8.8 million deaths in 2015. Unfortunately, the number of new cases is expected to rise by about 70% over the next two decades [39]. Moreover, the low response rate and tolerance are major concerns of current anti-cancer therapies [40–42]. Therefore, the development of complementary therapeutic interventions is desperately demanded. Danshen has been applied to treat cancerous disease in the history of TCM. One of its major active components, Tan II_A_, has showed promising effect in in vivo experiments and clinical studies [20, 36, 43]. The compound may influence cancer phenotypes through targeting multiple proteins. Hence the systematic approach developed in the present study fit to delineate the regulatory effects of Tan II_A_ on cancer-related pathways.

Recent advancements in computational technologies offered powerful tools that can be used to unravel the anticancer effect of Tan II_A_ in systematic format. Data mining and integration, large scale text-mining though ALS provided a comprehensive evidence-based set of protein targets of Tan II_A_. The visualization of interactions between the proteins via Cytoscape offered perceivable framework. Topological analysis on protein targets network of Tan II_A_ identified proteins that had significant influence on the network. However, Tan II_A_ possesses various pharmacological activities, cardio-protection and anticancer, for example, almost has nothing to do with each other. Therefore, topological analysis on a pack of proteins involved in various activities may somewhat fail to provide specific information. And the structure of topological clusters gathered by ClusterONE is generally susceptible to topological changes, thus the variations may not always represent pharmacological changes. Pharmacological clustering method used in the present study, however, could dramatically improve the robustness of clusters, the resulted anticancer network was a scale-free network with its node degree distribution followed power law, which guaranteed its good robustness and excellent error tolerance. Because power-law distribution implies that the majority of nodes have only a few links, nodes with small connectivity will be selected with much higher probability. The removal of these ‘small’ nodes does not alter the path structure of the remaining nodes, and thus has no impact on the overall communication effectiveness of the anticancer network [44]. Moreover, this approach helped to draw focus on the analysis of a specific bioactivity, take the anticancer effect for example. Further pathway analysis provided deep insight into five refined functional units of anticancer activity and shed new light on the molecular mechanisms of Tan II_A_. These mined targets data and pathway networks offered a systematic approach to unravel the anticancer mechanisms of Tan II_A_ and its effect on tumour microenvironment.

Tumour microenvironment describes the non-cancerous cells present in the tumour [45]. Increasing evidence suggests that the tumour microenvironment are quite complex, which includes fibroblasts and myofibroblasts, neuroendocrine cells, adipocytes, immunological and inflammatory cells, the blood and lymphatic vascular networks, and extracellular matrix [18]. Pharmacological clustering method identified the protective effects of Tan II_A_ against fibrosis diseases, inflammatory responses, and oxidative stress. These effects jointly play a positive role in improving tumour microenvironment.

The functional units of Tan II_A_ overlapped on signalling proteins, several proteins in signalling pathways, for instance MAPK signalling pathway and JAK/ STAT signalling pathway, were involved in multiple functional units and counted more than once. The large size (32 nodes) of the pathway network (Fig. 3) indicated that Tan II_A_ regulated multiple apoptosis pathways simultaneously and exerted its anticancer activity mainly through inhibiting anti-apoptosis of carcinoma cells. The MCC analysis suggested Bcl-2, Bcl-xL, and CHOP played key role in the network. These proteins are considered critical therapeutic targets of Tan II_A_ for anticancer therapy. The result revealed the importance of VEGF in sustained angiogenesis pathology. Tan II_A_ down-regulated this growth factor through four pathways and it showed highest degree in the network. It is notable that the pathway network suggested Tan II_A_ up-regulated PDGFR to promote vascular normalization therefore inhibited metastasis. The functional unit on carcinoma cell proliferation revealed that Tan II_A_ acted contrary to proteins in the same pathway, it inhibited PI3K/AKT, whereas induced the down-stream effector p27. The contradictory results proposed the possibility of a direct action of Tan II_A_ on p27. The inducible effect of Tan II_A_ on autophagic cell death was mainly executed by inhibition of mTOR pathway. The significance of mTOR was verified by its high degree of connection in the present pathway analysis.

In addition to the five functional units aforementioned, Tan II_A_ also regulated several other units that have not been illustrated for the sake of their limited size. It inhibited SMAD family member (Smad) 2 and 3 to attenuate insensitivity of cancer cell to anti-growth signals. The dedifferentiation of cancer cell was inhibited through inhibition of IL-6, CCAAT/enhancer binding protein alpha (CEBPα) [46], Spi-1 proto-oncogene (PU.1), c-Myc and E2F transcription factor 1 (E2F1). Moreover, Tan II_A_ inhibited NF-κB signalling and expression of MMP-2 and MMP-9, and increased levels of tissue inhibitor of matrix metalloproteinases type 1 and 2 (TIMP-1 and TIMP-2) [47], therefore suppressed invasion and metastasis of cancer cells.

The results of cell experiments announced the selectivity of Tan II_A_ on different cancer cell lines. It showed that HeLa and MDA-MB-231 were more sensitive to Tan II_A_ treatment than A549 and ACHN. Which confirmed the traditional uses of Danshen in gynaecology recorded in historical bibliographies such as The Great Pharmacopoeia and the Complete Book of Good Prescriptions for Women. Moreover, HepG2 was found to be sensitive to Tan II_A_ treatment as well. This finding also shed new light on the morden therapeutic use of Danshen on hepatic disorders.

## Conclusions

Cancer is one of the leading causes of death globally. Which has been recognized as a Systems Biology disease [48]. Therefore, a network-based approach has been applied in the present study to delineate the anticancer mechanism of Tan II_A_, the major active component of a Chinese medicinal herb: Danshen. We constructed pharmacological clustering method to focus network analysis on the pharmacological actions of Tan II_A_ instead of the topological features of network. Thereby the network analysis revealed that Tan II_A_ produced its anticancer effect mainly through induction of carcinoma cell apoptosis, cell cycle arrest and autophagic cell death; anti-proliferation and anti-angiogenesis. The study confirmed that Tan II_A_ systematically regulated molecular and protein events and cancer micro-environment to produce its anticancer effect. The cell experiments indicated the selectivity of Tan II_A_ towarded HeLa, HepG2 and MDA-MB-231 cell lines.

The present study offered an innovative approach for a comprehensive pathway study on the anticancer effect of Tan II_A_. The methodology constructed can be applied to a broad-spectrum of medicinal herbs and active compounds. It provided a novel researching method for actions of traditional Chinese medicinal herbs through protein networks in future. It focused not only on single target but also on several pathways that participated in the disease progression, and regulations of the herbal component on these pathways. As the concept of this approach is in consistent with the guiding theory of TCM that “the whole defines parts”, it proved to be a suitable method for TCM studies. The method offered guidance for further experiments which could hopefully minimize the suffering and sacrifices of experimental animals. As the current study mainly reveal the molecular mechanisms of Tan II_A_ through computational-based network pharmacological method, further experiments are still needed for confirming the regulatory effects of the mined protein targets and pathways.

## Methods

### Data mining

Cytoscape app “Agilent Literature Search” (ALS) software was used for data mining, it is a meta-search tool for automatically querying multiple text-based search engines (both public and proprietary) when a query was entered [49]. Query “Tanshinone II_A_” was entered, context was set to “blank”, maximum number of engine matches was set to 1000, interaction lexicon was set to “limited” and all other parameters were set to default. Obtained hits were checked against supporting evidences generated automatically during the search, false positives were removed.

### Pharmacological clustering

A clustering method, pharmacological clustering, based on physiological and/or pathological function of proteins identified by ALS was conducted. The clustering was based on information of genes and their coded protein which were gathered from NCBI databases and literatures. The anticancer module was obtained. The node degree distribution of anticancer network was analysed. Then proteins in the module were put into functional units according to ALS supporting evidences: cancer – apoptosis, cancer – proliferation, cancer – cell cycle, cancer – angiogenesis, cancer – differentiation and others. For instance, NCBI gene database indicated that BCL2 associated X (Bax) is involved in cancer cell apoptosis regulation, hence it was put in the pharmacological cluster cancer – apoptosis; whereas AKT serine/threonine kinase 1 (AKT), a component of PI3K/AKT signalling pathway, was included in several pharmacological clusters, such as cardio-protection and anti-inflammation, as evidences suggested that it is involved in various cellular processes. The clustering results were validated by PANTHER Classification System (Protein ANalysis THrough Evolutionary Relationships) [50] facilitated GO enrichment analysis. The protein specific UniProt ID of each target was obtained and processed for GO enrichment analysis for each cluster.

CytoHubba, a Cytoscape plug-in, was utilized to explore the important nodes/hubs in the ALS-constructed Tan II_A_ targets network on cancer. Matthews Correlation Coefficient (MCC) assessment was chosen as the ranking method, and ten top-ranking nodes were identified [51]. Other parameters were set to default values.

### Recognition and network construction of anticancer pathways regulated by Tan II_A_

The anticancer pathways regulated by Tan II_A_ were found by combining information obtained from KEGG and Cell Signalling Technology (CST) Pathways, the relations (induction and inhibition) between targets of Tan IIA were extracted. KEGG pathways “Pathways in Cancers (has 05200)”, “Apoptosis (has 04210)” and “Autophagy (hsa04140)”; CST pathways “Regulation of Apoptosis Overview”, “Autophagy Signalling”, “Angiogenesis”, “Cell Cycle Control: G1/S Checkpoint”, “PI3 Kinase/Akt Signalling”, “MAPK/Erk in Growth and Differentiation” and “mTOR Signalling” were used to delineate anticancer pathways regulated by Tan II_A_. Then the pathways were plotted using Cytoscape 3.5.1. The degree of a node in a pathway network was calculated by counting the number of connections it had.

### Cell proliferation evaluation by CCK-8 assay

*Cell culture* Human non-small-cell lung cancer cell line A549, human kidney cancer cell line ACHN, human hepatocellular carcinoma HepG2 and human triple-negative breast cancer cell line MDA-MB-231 were derived from ATCC and cultured in Dulbecco’s modified Eagle medium (DMEM; HyClone Laboratories, Logan, UT, USA) supplemented with 10% heat-inactivated fetal bovine serum (FBS; Gibco, Thermo Fisher Scientific, Shanghai, China) and 1% Penicillin-Streptomycin solution. Human cervical cancer cell line HeLa was cultured in RPMI-1640 supplemented with 10% FBS and 1% Penicillin-Streptomycin solution. The cell lines were incubated in a humidified atmosphere of 95% air and 5% CO_2_ at 37 °C. Tanshinone II_A_ (M.W. = 328, T4952, ≥97% -HPLC) was purchased from Jiangxi herbfine Co. Ltd. (CAS No. 568–72-9).

Cytotoxicity of Tan II_A_ was analysed by cell counting kit-8 (CCK-8; Biodee Biotechenology, Beijing, China) in A549, ACHN, MDA-MB-231, HepG2 and HeLa cell lines. The Dojindo’s highly water-soluble tetrazolium salt WST-8 [2-(2-methoxy-4-nitrophenyl)-3-(4-nitrophenyl)-5-(2,4-disulfophenyl)-*2H*-tetrazolium, monosodium salt] contained in CCK-8 solution produces a water-soluble formazan orange colour dye upon reduction. The assay is based on this reduction of WST-8 by dehydrogenases in living cells. The amount of the formazan dye generated is directly proportional to the number of living cells. Cells for CCK8 assay were seeded in a 96-well-microplate at a density of 3600 cells/well and treated with various concentrations of tanshinone II_A_ for 48 h. Control groups were treated with 10 μM doxorubicin at the same incubation environment for 48 h. After the treatment, CCK-8 solution (10 μL) was added to each well, the plate was then incubated for 2 h before the absorbance was measured at 450 nm using a microplate reader.

### Statistical analyses

The in vivo experiment data were presented as mean ± standard deviation. The experiments were conducted independently for at least three times. Dose-response curves and IC_50_ values were drawn and calculated by Graphpad prism (version 5, GraphPad Software, Inc., Carlifonia).

## Additional file

Additional file 1: Tanshinone II A anti-cancer targets pool. (DOCX 16 kb)

## Abbreviations

Akt: protein kinase B
ALS: Agilent Literature Search
ATF4: activating transcription factor 4
Bax: BCL2 associated X
Bcl-xL: B-cell lymphoma-extra large
BECN1: beclin-1
BIP: heat shock protein family A member 5
CCND1: cyclin D1
CDC2: cyclin dependent kinase 1
CEBPα: CCAAT/enhancer binding protein alpha
CHOP: DNA damage inducible transcript 3
c-Myc: MYC proto-oncogene
CST: Cell Signaling TECHNOLOGY
DR5: TNF receptor superfamily member 10b
E2F1: E2F transcription factor 1
EGFR: epidermal growth factor receptor
ERK: extracellular signal–regulated kinases
Fas: Fas cell surface death receptor
Fos: Fos proto-oncogene, AP-1 transcription factor subunit
HER2: erb-b2 receptor tyrosine kinase 2
IAP: inhibitor-of-apoptosis protein
IGFR: insulin-like growth factor 1 receptor
IL-8: interleukin-8
JAK: Janus Kinase
JNK: c-jun N-terminal kinase
Maspin: mammary serine protease inhibitor
MCL1: induced myeloid leukemia cell differentiation protein
MEK: mitogen-activated protein kinase kinase
MMP2: matrix metalloproteinase 2
mTOR: mechanistic target of rapamycin
NCBI: National Center for Biotechnology Information
NF-κB: nuclear factor kappa B
NO: nitric oxide
NRF2: nuclear factor (erythroid-derived 2)-like 2
p16: cyclin-dependent kinase inhibitor 2A
p21: cyclin-dependent kinase inhibitor 1
p27: interferon alpha-inducible protein 27
p38 MAPK: p38 mitogen-activated protein kinase
p70 S6K: ribosomal protein S6 kinase B1
PARP1: poly(ADP-ribose) polymerase 1
PDGRF: platelet derived growth factor receptor
PI3K: phosphoinositide 3-kinase
PU.1: Spi-1 proto-oncogene
Smad: SMAD family member
STAT: Signal Transducer and Activator of Transcription
Tan II_A_: Tanshinone II_A_
TCM: traditional Chinese medicine
TCTP: translationally controlled tumour protein
TIMP-1: matrix metalloproteinases type 1
TIMP-2: matrix metalloproteinases type 2
TRAF: TNF receptor associated factor
VEGF: vascular endothelial growth factor
