# Supplementary material for: The anticancer mechanism investigation of Tanshinone IIA by pharmacological clustering in protein network
Source: BMC Syst Biol. 2018 Oct 29;12:90. doi: 10.1186/s12918-018-0606-6 (PMC6206912; doi:10.1186/s12918-018-0606-6)
Supplement: Supplementary file 1 — Tanshinone II A anti-cancer targets pool. (DOCX 16 kb) [file 12918_2018_606_MOESM1_ESM.docx]

**Supporting evidence**

Tanshinone Ⅱ_A_ targets pool

1. Li, G. et al. Tanshinone IIA Inhibits HIF-1 α and VEGF Expression in Breast Cancer Cells via mTOR / p70S6K / RPS6 / 4E-BP1 Signaling Pathway. PLoS One 1, 1–14 (2015).

2. Xie, J. et al. The antitumor effect of tanshinone IIA on anti- proliferation and decreasing VEGF / VEGFR2 expression on the human non-small cell lung cancer A549 cell line. Acta Pharm. Sin. B 5, 554–563 (2015).

3. Jiao, J. & Wen, F. Tanshinone IIA acts via p38 MAPK to induce apoptosis and the down-regulation of ERCC1 and lung-resistance protein in cisplatin-resistant ovarian cancer cells. Oncol. Rep. 781–788 (2011). doi:10.3892/or.2010.1107

4. Pan, T., Wang, P., Hung, Y., Huang, C. & Rau, K. Proteomic analysis reveals tanshinone IIA enhances apoptosis of advanced cervix carcinoma CaSki cells through mitochondria intrinsic and endoplasmic reticulum stress pathways. Proteomics 13, 3411–3423 (2013).

5. Chen, F. et al. Biomaterials Science and anti-hepatocellular carcinoma e ffi cacy of. Biomater. Sci. (2015). doi:10.1039/c5bm00224a

6. Liu, F. et al. An NQO1-Initiated and p53-Independent Apoptotic Pathway Determines the Anti-Tumor Effect of Tanshinone IIA against Non-Small Cell Lung Cancer. PLoS One 7, e42138 (2012).

7. Shan, Y. et al. Inhibitory effects of tanshinone II-A on invasion and metastasis of human colon carcinoma cells. Acta Pharmacol. Sin. 1537–1542 (2009). doi:10.1038/aps.2009.139

8. Yuxian, X., Feng, T., Ren, L. & Zhengcai, L. Tanshinone II-A inhibits invasion and metastasis of human hepatocellular carcinoma cells in vitro and in vivo. Tumori 95, 789–795 (2009).

9. Lin, C. et al. Tanshinone IIA Inhibits Breast Cancer Stem Cells Growth In Vitro and In Vivo Through Attenuation of IL‐6/STAT3/NF‐kB Signaling Pathways. J. Cell. Biochem. 114, 2061–2070 (2013).

10. Tu, J. et al. International Immunopharmacology TanshinoneIIA ameliorates in fl ammatory microenvironment of colon cancer cells via repression of microRNA-155. Int. Immunopharmacol. 14, 353–361 (2012).

11. Zhang, H. et al. Tanshinone Ⅱ A inhibits human esophageal cancer cell growth through. Arch. Biochem. Biophys. 598, 50–56 (2016).

12. Ma, Z., Zhang, B., Wang, D., Li, X. & Wei, J. Tanshinones suppress AURKA through up-regulation of miR-32 expression in non-small cell lung cancer. Oncotarget. 6, 20111–120

13. Huang, C. Y. E. N., Chiu, T. L. & Kuo, S. J. E. N. Tanshinone IIA inhibits the growth of pancreatic cancer BxPC ‑ 3 cells by decreasing protein expression of TCTP , MCL ‑ 1 and Bcl ‑ xL. Mol. Med. Rep. 7, 1045–1049 (2013).

14. Su, C. & Lin, Y. Tanshinone IIA down-regulates the protein expression of ErbB-2 and up-regulates TNF- α in colon cancer cells in vitro and in vivo. Int. J. Mol. Med. 22, 847–851 (2008).

15. Wang, L. et al. Blocking of JB6 Cell Transformation by Tanshinone IIA : Epigenetic Reactivation of Nrf2 Antioxidative Stress Pathway. AAPS J. 16, 1214–1225 (2014).

16. Pan, T., Hung, Y., Pei-wen, W., Chen, S. & Hsu, T. Functional proteomic and structural insights into molecular targets related to the growth inhibitory effect of tanshinone IIA on HeLa cells. Proteomics 10, 914–929 (2010).

17. Paper, O. Growth Inhibition and Apoptosis Induction by Tanshinone IIA in Human Colon Adenocarcinoma Cells. Planta Med 74, 1357–1362 (2008).

18. Chang, C., Kuan, C., Lin, J., Lai, J. & Ho, T. Tanshinone IIA Facilitates TRAIL Sensitization by Up-regulating DR5 through the ROS-JNK-CHOP Signaling Axis in Human Ovarian Carcinoma Cell Lines. Chem.Res.Toxicol. 28, 1574–1583 (2015).

19. Kim, E. et al. Tanshinone IIA induces TRAIL sensitization of human lung cancer cells through selective ER stress induction. Int. J. Oncol. 48, 2205–2212 (2016).

20. Su, C. C. Tanshinone IIA inhibits human gastric carcinoma AGS cell growth by decreasing BiP , TCTP , Mcl ‑ 1 and Bcl ‑ xL and increasing Bax and CHOP protein expression. Int. J. Mol. Med. 34, 1661–1668 (2014).

21. Chiu, S. C. et al. Tanshinone IIA inhibits human prostate cancer cells growth by induction of endoplasmic reticulum stress in vitro and in vivo. Prostate Cancer Prostatic Dis. 16, 315–322 (2013).

22. Su, C. Tanshinone IIA Inhibits Gastric Carcinoma AGS Cells Through Increasing p-p38 , p-JNK and p53 but Reducing p-ERK , CDC2 and Cyclin B1 Expression. Anticancer Res. 34, 7097–7110 (2014).

23. Tang, C., Xue, H., Huang, H. & Wang, X. Tanshinone IIA inhibits constitutive STAT3 activation , suppresses proliferation , and induces apoptosis in rat C6 glioma cells. Neurosci Lett. 470, 126–129 (2010).

24. Huang, X., Li, Y., Li, J., Feng, Y. & Xu, X. Tanshinone IIA dampens the cell proliferation induced by ischemic insult in rat astrocytes via blocking the activation of HIF-1 α / SDF-1 signaling. Life Sci. 112, 59–67 (2014).

25. Li, C., Han, X., Zhang, H., Wu, J. & Li, B. The interplay between autophagy and apoptosis induced by tanshinone IIA in prostate cancer cells. Tumor Biol. 1–8 (2015). doi:10.1007/s13277-015-4602-9

26. Liu, J., Zhang, Y., Lin, D. & Xiao, R. Tanshinone IIA inhibits leukemia THP-1 cell growth by induction of apoptosis. Oncol. Rep. 21, 1075–1081 (2009).

27. Liu, J., Lin, D., Liu, P., Huang, M. & Li, X. Induction of apoptosis and inhibition of cell adhesive and invasive effects by tanshinone IIA in acute promyelocytic leukemia cells in vitro. J. Biomed. Sci. 13, 813–823 (2006).

28. Chien, S., Kuo, S., Chen, Y. & Chen, D. Tanshinone IIA inhibits human hepatocellular carcinoma J5 cell growth by increasing Bax and caspase 3 and decreasing CD31 expression in vivo. Mol. Med. Rep. 5, 282–286 (2012).

29. Gao, H. et al. Total Tanshinones-Induced Apoptosis and Autophagy Via Reactive Oxygen Species in Lung Cancer 95D Cells. Am. J. Chinese Med. 43, 1–15 (2015).

30. Yun, S. et al. Tanshinone IIA Induces Autophagic Cell Death via Activation of AMPK and ERK and Inhibition of mTOR and p70 S6K in KBM-5 Leukemia Cells. Phyther. Res. 28, 458–464 (2014).

31. Wang, J., Feng, J., Han, J., Zhang, B. & Mao, W. The Molecular Mechanisms of Tanshinone IIA on the Apoptosis and Arrest of Human Esophageal Carcinoma Cells. Biomed Res. Int. 1–9 (2014). doi:10.1155/2014/582730

32. Su, C. & Chiu, T. Tanshinone IIA decreases the protein expression of EGFR , and IGFR blocking the PI3K / Akt / mTOR pathway in gastric carcinoma AGS cells both in vitro and in vivo. Oncol. Rep. 36, 1173–1179 (2016).

33. Chan, S. et al. Effect of Supplementation of Tanshinone IIA and Sodium Tanshinone IIA Sulfonate on the Anticancer Effect of Epirubicin : An In Vitro Study. Evid Based Complement Altern. Med. 1–9 (2011). doi:10.1155/2011/841564

34. Won, S., Lee, H., Jeong, S. & Lee, H. Tanshinone IIA Induces Mitochondria Dependent Apoptosis in Prostate Cancer Cells in Association with an Inhibition of Phosphoinositide 3- Kinase / AKT Pathway. Biol. Pharm. Bull. 33, 1828–1834 (2010).

35. Bai, Y., Zhang, L., Fang, X. & Yang, Y. Tanshinone IIA enhances chemosensitivity of colon cancer cells by suppressing nuclear factor- κ B. Exp. Ther. Med. 11, 1085–1089 (2016).

36. Shan, Y. et al. Inhibitory effects of tanshinone II-A on invasion and metastasis of human colon carcinoma cells. Acta Pharmacol. Sin. 30, 1537–1542 (2009).

37. Xing, Y., Tu, J., Zheng, L., Guo, L. & Xi, T. Anti-angiogenic effect of tanshinone IIA involves inhibition of the VEGF / VEGFR2 pathway in vascular endothelial cells. Oncol. Rep. 33, 163–170 (2015).

38. Jung, J. H. et al. Apoptosis Induced by Tanshinone IIA and Cryptotanshinone Is Mediated by Distinct JAK / STAT3 / 5 and SHP1 / 2 Signaling in Chronic Myeloid Leukemia K562 Cells. Evid Based Complement Altern. Med. 1–10 (2013).

39. Jin, Y. et al. Inhibition of cytochrome P450 2J2 by tanshinone IIA induces apoptotic cell death in hepatocellular carcinoma HepG2 cells. Eur. J. Pharmacol. 764, 480–488 (2015).

40. Tsai, M., Yang, R., Wu, H., Pang, J. S. & Huang, S. Anti-angiogenic effect of Tanshinone IIA involves inhibition of matrix invasion and modification of MMP-2 / TIMP-2 secretion in vascular endothelial cells. Cancer Lett. 310, 198–206 (2011).

41. Yun, S. et al. Activation of c-Jun N-Terminal Kinase Mediates Tanshinone IIA-Induced Apoptosis in KBM-5 Chronic Myeloid Leukemia Cells. Biol. Pharm. Bull. 36, 208–214 (2013).

42. Wang, W. et al. Tanshinone IIA inhibits metastasis after palliative resection of hepatocellular carcinoma and prolongs survival in part via vascular normalization. J. Hematol. Oncol. 5, 1–11 (2012).

43. Zhang, Z. et al. Tanshinone IIA triggers p53 responses and apoptosis by RNA polymerase II upon DNA minor groove binding. Biochem. Pharmacol. 78, 1316–1322 (2009).

44. Munagala, R. et al. Tanshinone IIA inhibits viral oncogene expression leading to apoptosis and inhibition of cervical cancer. Cancer Lett. 356, 536–546 (2015).

45. Won, S., Lee, H., Jeong, S., Lü, J. & Kim, S. Activation of p53 Signaling and Inhibition of Androgen Receptor Mediate Tanshinone IIA Induced G1 Arrest in LNCaP Prostate Cancer Cells. Phyther. Res. 26, 669–674 (2012).
